# Optimal nasotracheal tube insertion depth in neonates

**DOI:** 10.3389/fped.2026.1770644

**Published:** 2026-02-24

**Authors:** Susanne Tippmann, Martin Haan, Eva Mildenberger, Dirk Wackernagel, André Kidszun

**Affiliations:** 1 Department of Neonatology, University Medical Centre of the Johannes Gutenberg-University Mainz, Mainz, Germany; 2 Division of Neonatology, Department of Pediatrics, Inselspital, Bern University Hospital, University of Bern, Bern, Switzerland

**Keywords:** endotracheal tube insertion depth, intubation, nasotracheal intubation, neonates, preterm

## Abstract

**Aim:**

Existing recommendations for nasotracheal endotracheal tube (ETT) insertion depth in neonates have shown remarkable consistency over decades and have recently been prospectively evaluated in clinical practice. However, large prospective datasets systematically validating biometric predictors and quantifying expected variability remain limited. This study aimed to confirm established nasotracheal ETT depth recommendations using a large prospective cohort and to translate these findings into a standardized, evidence-based bedside reference.

**Methods:**

We analyzed 497 nasotracheal intubations performed between 2017 and 2023 in a tertiary neonatal intensive care unit. Tube position was prospectively assessed after each intubation using standardized chest radiography. Optimal ETT placement was defined as the tube tip located between the clavicles and at least 1 cm above the tracheal carina. Clinical and biometric parameters were systematically recorded, and their associations with insertion depth were evaluated using LOESS curves and linear regression models.

**Results:**

Across a wide range of gestational ages and body weights, body weight at the time of intubation showed the strongest and most consistent association with optimal nasotracheal ETT insertion depth (adjusted R^2^ = 0.88; RMSE = 0.52). Based on these findings, an evidence-based chart with defined tolerance ranges and a complementary web-based decision-support tool were developed to facilitate standardized bedside estimation.

**Conclusion:**

In this large prospective cohort, body weight at the time of intubation was confirmed as the most reliable single predictor of optimal nasotracheal endotracheal tube insertion depth in neonates. Our findings support established reference ranges and provide quantitative confirmation across a broad spectrum of gestational ages and body weights. By translating these data into a concise, evidence-based bedside chart and a complementary digital reference, this study strengthens confidence in existing recommendations and supports standardized clinical practice, while emphasizing the need for clinical judgement and post-intubation verification.

## 1 Introduction

Neonatal intubation, particularly in preterm and critically ill infants, is a technically demanding and time critical-procedure that requires precise airway management. A meta-analysis by Razak et al. found that more than 50% of neonates born before 30 weeks' gestation had malpositioned endotracheal tubes (ETTs) (1). Incorrect ETT insertion depth can lead to complications such as atelectasis, pneumothorax, airway trauma, and accidental extubation (2,3). Malpositioned ETTs may also necessitate repositioning and increase radiation exposure due to repeated imaging, all of which compromise patient safety and comfort. In emergency or transport settings - where x-ray confirmation may not be feasible - accurate ETT placement becomes even more demanding. Moreover, optimal positioning is essential for effective and safe surfactant administration (4).

While several guidelines exist to estimate ETT insertion depth, most are based on small cohorts and focus primarily on orotracheal intubation. Nasotracheal intubation, although commonly used in many neonatal intensive care units (NICUs), is less well supported by precise, data-driven recommendations. A variety of studies and efforts have already been made to evaluate the safety of the correct ETT insertion depth, especially in cases where x-ray evaluating is delayed, for example during neonatal transport. Bellini et al. proceeded to develop a nomogram, which, in its subsequent evolution as the Genoa Formula, has gained application in the context of very small premature infants (5,6). The European Resuscitation Council (ERC) suggests in 2021 adding approximately 1 cm to oral intubation depths for nasal intubation, but this generic adjustment lacks precision, particularly in extremely preterm infants (7). The current guideline from 2025 now refers to a retrospective study by Maiwald et al. and makes recommendations for oral and nasal tube insertion depth based on birth weight and gestational age. (8,9) Traditional estimation methods like Tochen's rule have shown limited accuracy, especially in neonates with very low birth weight (10–12).

This study aimed to confirm established nasotracheal ETT depth recommendations using a large prospective cohort and to translate these findings into a standardized, evidence-based bedside reference.

## 2 Methods

This observational study was performed between May 2017 and August 2023 at a tertiary care NICU of the University Medical Centre Mainz. Nasotracheal intubation was the standard approach in this unit. The study was conducted as part of a broader analysis of adverse events associated with neonatal intubation (13). Data were collected prospectively using standardized documentation forms, while model development and analysis were performed retrospectively. Each intubation was counted as a new event, even if a patient underwent multiple intubations during the study period.

Following each intubation, a standardized chest x-ray (anteriorposterior, with a focus-to-film distance of 100 cm) in a neutral head position was obtained to verify correct ETT placement. Neutral head position was defined as and the infants' head placed in the midline sniffing position, as it is described in the ERC recommendation for opening a newborn's airway during resuscitation (8). If repositioning was required, the adjustment was documented and, when necessary, confirmed with a follow-up x-ray. Each intubation was recorded using a standardized data sheet that included demographic and biometric information: gestational age, birth weight, birth length, head circumference, as well as postmenstrual age, and weight, length, head circumference, nasal-tragus length, and tragus-to-xiphoid distance at the time of intubation. Nasal–tragus length and tragus-to-xiphoid distance were included as exploratory anthropometric parameters based on prior studies suggesting a relationship between external body landmarks and airway length (14). These measurements were collected to assess their potential value as they might be easy to assess in situations where body weight or gestational age are unavailable, such as in emergency or transport settings. Z-scores for anthropometric parameters were calculated using the Fenton growth charts (15). Neonates were categorized as small (SGA), appropriate (AGA), or large (LGA) for gestational age if their measurements were more than ±2 standard deviations from population norms at birth. We also applied this categorization to the time of intubation.

**Figure 1:**
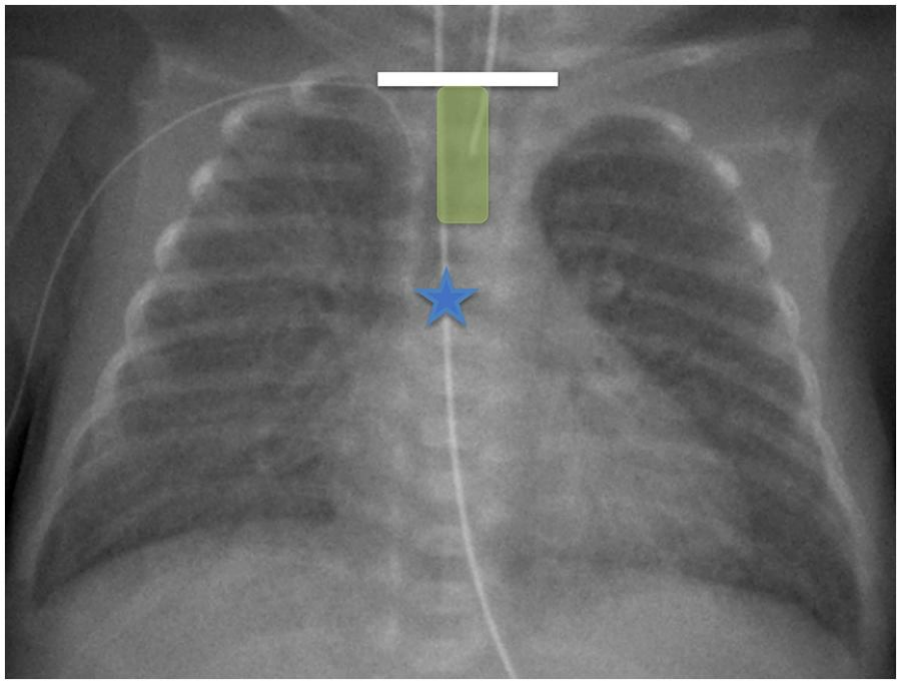
Chest x-ray of a preterm infant at 26 weeks’ gestation illustrating correct nasotracheal ETT placement. The white bar indicates the imagined line between the clavicles, the blue star marks the tracheal carina, and the green bar denotes the optimal tube position between these anatomical landmarks.

The optimal ETT tip position was defined as placement between the midpoint of the imagined line between the clavicles and at least 1 cm above the tracheal carina (Figure 1). The attending neonatologist assessed positioning based on x-ray evaluation as the clinical reference standard, acknowledging the inherent individual and technical variability.

For data analysis, scatter plots were created and smoothed using locally estimated scatterplot smoothing (LOESS) curves. Linear regression models were constructed using the lm() function in R. The gestational age–based model was refined by excluding measurements from SGA and LGA neonates. Similarly, anthropometric models were optimized by removing data points outside the ±2 Z-score range at the time of intubation. Model performance was compared using the compare package (v0.10.9, CRAN).

Descriptive statistics were presented as median and interquartile range (IQR) for continuous variables, and as absolute numbers and percentages for categorical data. All analyses were conducted using R (version 4.4.0; R Foundation for Statistical Computing, Vienna, Austria). Packages used included ggplot2 (v3.4.4) for visualization, gtsummary (v1.7.2) for summary statistics, and performance for model evaluation. A *p*-value < 0.05 was considered statistically significant.

Based on the regression equations, we developed a web-based application that allows users to enter available biometric data. The model prioritizes current weight as the most predictive factor; if weight is unavailable, predictions are based on other variables in descending order of predictive performance: body length, head circumference, postmenstrual age, tragus-to-xiphoid distance, and nasal-tragus length. Predicted insertion depth is given in centimeters with one decimal place.

In addition to the application, we created a figure illustrating the predicted insertion depth (with standard error) based on the weight model, as well as a table that offers recommended depths based on weight and postmenstrual age.

This study is registered in the German Clinical Trial Register under reference number DRKS00013575 (16). As it involved the analysis of routine parameters, consent to utilize the data was not required. Approval for this study was given by the Ethics Committee of the Rhineland-Palatinate Medical Board on 7 September 2017 under review number 837.397.17 (11231).

## 3 Results

### 3.1 Study population

A total of 497 nasotracheal intubations were analyzed. Gestational age at birth ranged from 22 + 0 to 42 + 5 weeks, and birth weight varied between 400 g and 5030 g. At the time of intubation, the postmenstrual age ranged from 22 + 0 to 45 + 0 weeks. Detailed characteristics of the study cohort are presented in Table 1.

**Table 1:** Demographic and clinical characteristics of the study cohort.

Characteristic	*N* = 497^a^
GA [weeks]	29.0 (25.7, 34.4)
Birth weight [g]	1,120 (720, 2,340)
Missing data	2
Anthropometry	
SGA	22 (4.5%)
AGA	461 (94%)
LGA	9 (1.8%)
Missing data	5
Sex	
female	213 (43%)
male	284 (57%)
PMA [weeks] (intubation)	30.1 (26.7, 35.3)
Missing data	1
Weight [g] (intubation)	1,280 (780, 2,420)
Missing data	2
Length [cm] (intubation)	39 (33, 47)
Missing data	34
Head circumference [cm] (intubation)	27.0 (23.5, 32.0)
Missing data	36
Intubation on DOL	
DOL 1	303 (61%)
DOL 2-7	64 (13%)
DOL >7	129 (26%)
Missing data	1

^a^ Median (Q1, Q3); *n* (%), *N* = Intubations.

In 140 cases, the initial ETT insertion depth required correction (140/497, 28.2%). The amplitude of the corrections exhibited a range from 0.2 to 1.7 centimeters (mean 0.34 centimeters). In the majority of cases, the tube was withdrawn, indicating that it had initially been placed too deeply.

Final ETT insertion depth was available for correlation with several biometric parameters, including body weight at the time of intubation (*n* = 495), postmenstrual age (*n* = 496), body length (*n* = 463), head circumference (*n* = 461), nasal–tragus length (*n* = 281), and tragus–to-xiphoid distance (*n* = 219).

### 3.2 Association between biometric parameters and insertion depth

**Figure 2:**
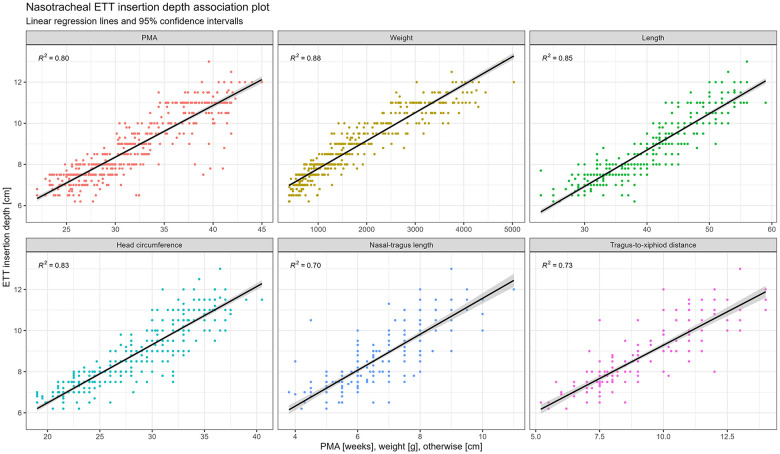
Linear regression models predicting nasotracheal ETT insertion depth based on biometric parameters. Each model includes a 95% confidence interval for the regression line. Optimized models were derived by excluding outliers outside the ±2 Z-score range.

All assessed biometric parameters showed a positive association with final ETT insertion depth. LOESS smoothing demonstrated an approximately linear relationship across the observed clinical range (Supplementary Figure S1). Based on these associations, linear regression models were developed for all parameters (Figure 2).

To optimize model performance, specific exclusions were applied. In the postmenstrual age (PMA)-based model, neonates classified as small or large for gestational age (SGA and LGA) were excluded (*n* = 31, 6.3%). Through this the association increased from R^2^ = 0.802 to R^2^ = 0.851. For the weight, length, and head circumference models, values outside the ±2 standard deviation range based on Fenton growth references were removed. This resulted in the exclusion of 31 cases (6.3%) for weight, 54 cases (12%) for length, and 53 cases (11.5%) for head circumference.

Among all evaluated parameters, body weight at the time of intubation showed the strongest association with optimal insertion depth (adjusted R^2^ = 0.883; RMSE = 0.494). Performance metrics for all models are provided in Supplementary Table S1.

### 3.3 Web application

A web-based application (http://www.optupos.com) was developed to translate the regression models into a clinically accessible format. The application generates independent predictions based on individual biometric parameters. When multiple parameters are entered, the output is based on the parameter with the highest predictive performance, rather than on a combined multivariable estimate.

Users may enter any available biometric data, including weight, gestational age, body length, head circumference, nasal–tragus length, and tragus–to-xiphoid distance. When body weight is provided, the prediction is based on the weight model; if unavailable, the application automatically selects the parameter with the next highest predictive performance. Individual parameter-specific predictions can also be viewed to support clinical decision-making, particularly when results deviate from clinical expectations.

Predicted insertion depths are displayed in centimeters to one decimal place. The application is available in both English and German and is optimized for use on smartphones and desktop devices. The tool is based on the data presented in this study and has not yet undergone prospective clinical validation. A demonstration video is provided in the Supplementary Material.

### 3.4 Recommendation chart

In addition to the web application, a static graph and lookup table were developed as alternative resources for situations in which digital tools are unavailable. These resources are based on the weight- and PMA-based models and provide recommended insertion depths in half-centimeter increments.

**Figure 3:**
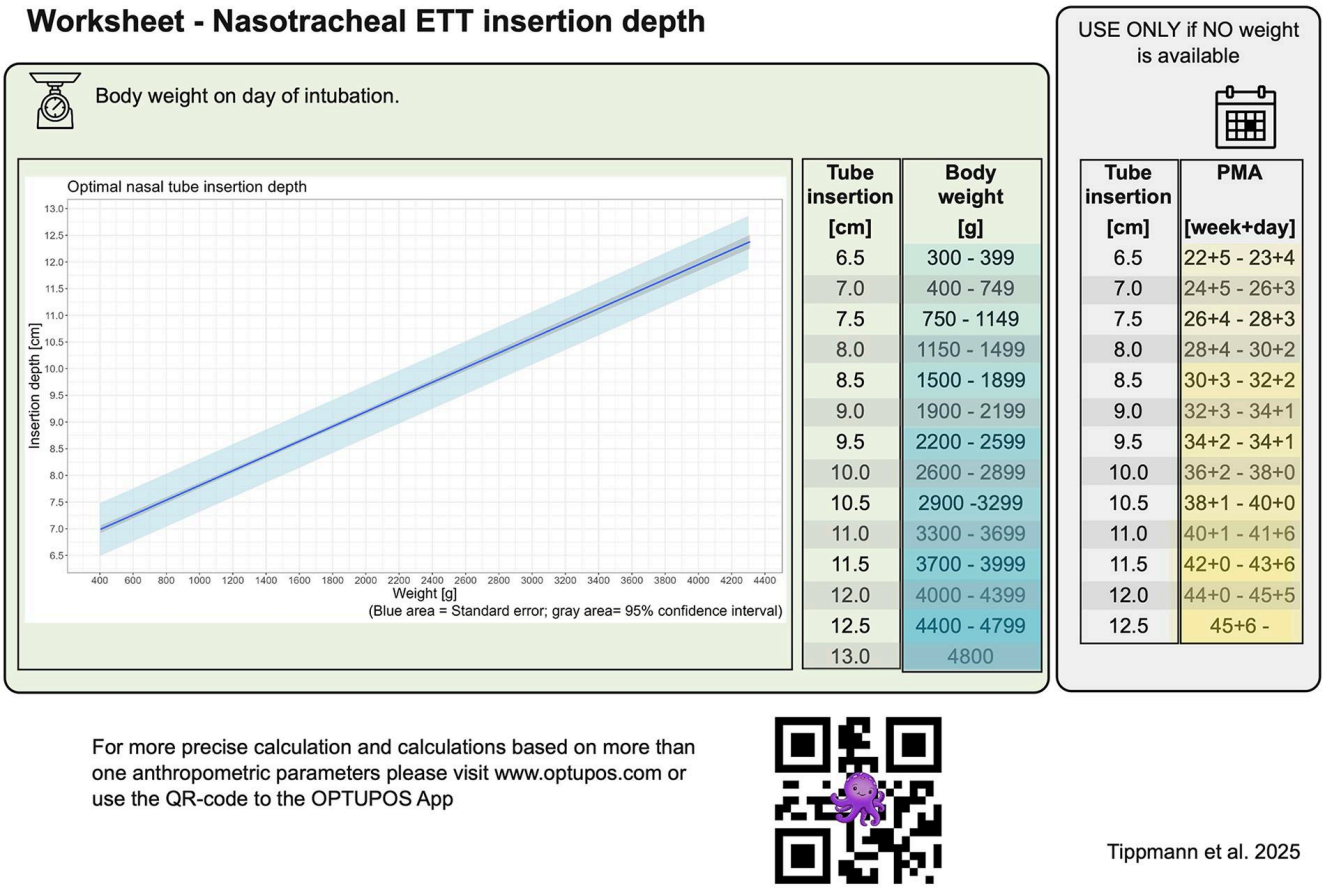
Graph depicting predicted nasotracheal ETT insertion depth (*y*-axis) in relation to weight (*x*-axis), based on the optimized regression model. The blue shaded area represents the standard error range. This visualization supports bedside estimation when the digital tool is unavailable.

The graph includes a visual representation of the standard error, illustrating a tolerance range to support rapid estimation in emergency settings. While offering lower resolution than the dynamic web application, these tools allow clinically acceptable estimation of nasotracheal ETT insertion depth when time or technical access is limited (Figure 3).

## 4 Discussion

In this large cohort of nearly 500 prospectively collected intubation routine data, body weight at the time of intubation emerged as the most consistent single predictor of optimal nasotracheal endotracheal tube insertion depth in neonates. Based on these findings, we developed a bedside reference tool that translates established estimation principles into a data-driven format with improved usability compared with traditional static rules or tables. The resulting web application and accompanying chart support individualized clinical decision-making while remaining within clinically relevant safety margins. Across a broad range of gestational ages and body weights, the observed relationships were largely linear and in line with previously published reference ranges. Rather than introducing fundamentally new insertion depths, our findings provide quantitative confirmation of existing recommendations and support their applicability in contemporary neonatal practice.

Previous work has demonstrated that even minor deviations in ETT insertion depth may be associated with clinically relevant respiratory complications. Murphy et al. highlighted that minor depth differences can influence ventilation outcomes, particularly in vulnerable preterm infants (17). In this context, it is reassuring that recent European Resuscitation Council guidelines incorporate recommendations derived from retrospective analyses by Maiwald et al., which demonstrated a strong correlation between optimal ETT position and body weight based on radiographic evaluation (8,9). In that study, 162 chest x-rays were analyzed, and the proposed approach has subsequently been evaluated prospectively (18). Ebenebe et al. similarly reported a weight-based regression model with improved performance compared with historical estimation methods (19). Although derived from different cohorts and study designs, the resulting recommended insertion depths across these studies are remarkably consistent and could be compared with our data in a future analysis.

Furthermore, Bellini et al. introduced a nomogram-based strategy derived from anatomical reference points, with particular relevance for emergency situations and neonatal transport settings (5). Their work underscores that, despite methodological differences, the range of clinically acceptable insertion depths is inherently narrow, especially in very preterm and extremely low birth weight infants. This concept is supported by subsequent studies reporting that different estimation methods - whether based on body weight, gestational age, or external biometric landmarks - tend to converge within a small margin of variation when correct tube positioning is achieved (14, 20). In addition, Takeuchi et al. demonstrated in a small cohort that body weight remained the strongest predictor of insertion depth even in preterm infants weighing less than 750 g, whereas gestational age showed reduced predictive accuracy when small-for gestational-age infants were included (12).

Comparison with prenatal biometric data also offers interesting insights. Selter et al. demonstrated that prenatal measurements can predict ETT insertion depth with reasonable accuracy (21). However, their approach requires precise fetal biometric data and strong interdisciplinary collaboration between neonatologists and obstetricians. In emergency situations, obtaining these measurements may not be feasible, limiting the widespread applicability of this method. Additionally, the efficacy of these methods is primarily confined to delivery room intubations.

Other pragmatic strategies, such as using the black depth markings on ETTs, offer rapid visual guidance but are prone to inaccuracy, particularly in extremely low birth weight infants, in whom airway proportions differ substantially (22). Wang et al. evaluated nasal–tragus length as an external biometric surrogate and found improved accuracy in a Taiwanese cohort (14). Nevertheless, this approach requires precise measurement technique and training and may be difficult to apply reliably in acute clinical settings.

In recognition of real-world challenges, we developed a digital bedside usable tool (http://www.optupos.com) and simplified table and graph derived from our dataset. These resources use half-centimeter depth ranges that align with clinical measurement conventions and are designed for easy application in urgent situations. The non-linear weight groupings in these tools reflect the observed distribution of safe insertion depths. Importantly, the application does not generate combined multivariable predictions; instead, it prioritizes the single parameter with the highest predictive performance, weight at time of intubation. The tools are designed to support, rather than replace, clinical judgement and established verification methods by providing an evidence-based point of reference within accepted safety margins.

Several limitations should be acknowledged. Although data collection was prospective, the predictive models and tools have not yet been prospectively validated in real-time clinical use. Exclusion of small- and large-for-gestational-age neonates improved model performance but may limit applicability in these subgroups. The extent of these slight restraints merits particular consideration in a prospective analysis. Chest radiography was used as the clinical reference standard for tube position, recognizing inherent anatomical and technical variability, including divergences in patient positioning and tube fixation. Importantly, the reference range used to define optimal endotracheal tube position in the present study was deliberately broader than in those referenced. Finally, while predictions are reported to one decimal place, the clinical relevance of millimeter-level differences remains limited by biological variability and practical fixation tolerances. External validation in independent neonatal populations will be essential to further assess generalizability.

## 5 Conclusion

In this large cohort of prospectively collected routine neonatal intubations, body weight at the time of intubation was confirmed as the most reliable single predictor of optimal nasotracheal endotracheal tube insertion depth. Our findings corroborate existing weight- and gestational age–based recommendations and provide quantitative support across a broad clinical range. By translating these data into a web application as digital reference tool and an evidence-based bedside chart, this study supports standardized and clinically pragmatic decision-making while emphasizing the continued importance of clinical judgement and post-intubation verification.

Future studies should aim to validate this model in external neonatal populations and assess its integration into clinical workflows and digital decision-support systems. Expanding the tool's use to include orotracheal intubation may further broaden its applicability in neonatal care.

## Data Availability

The datasets generated during and/or analyzed during the current study are available from the corresponding author on reasonable request.

## Supplementary material

Supplementary Figure S1 Scatter plots showing the relationship between final nasotracheal ETT insertion depth and biometric parameters: postmenstrual age (PMA), weight, length, head circumference, nasal-tragus length, and tragus–to–xiphoid distance. LOESS smoothing curves illustrate the overall trends

Supplementary Video 1 The web application offers the option of entering all available biometric data (weight, gestational age, head circumference, length, nasal-tragus length, tragus to xiphoid distance). Once entered, the recommended insertion depth is displayed in centimeters, calculated based on weight, if entered. As illustrated in the chart, the calculated insertion depth for the respective biometric data is shown as a turquoise bar, with its predictive power indicated by the grey bar. On the start page, users have the option to modify any entered data or to initiate a new intubation. The input mask is available in both English and German, and can be changed in the top left-hand corner.
