# Supplementary figures and images for: Optimal nasotracheal tube insertion depth in neonates
Source: Front Pediatr. 2026 Feb 24;14:1770644. doi: 10.3389/fped.2026.1770644 (PMC12971967; doi:10.3389/fped.2026.1770644)

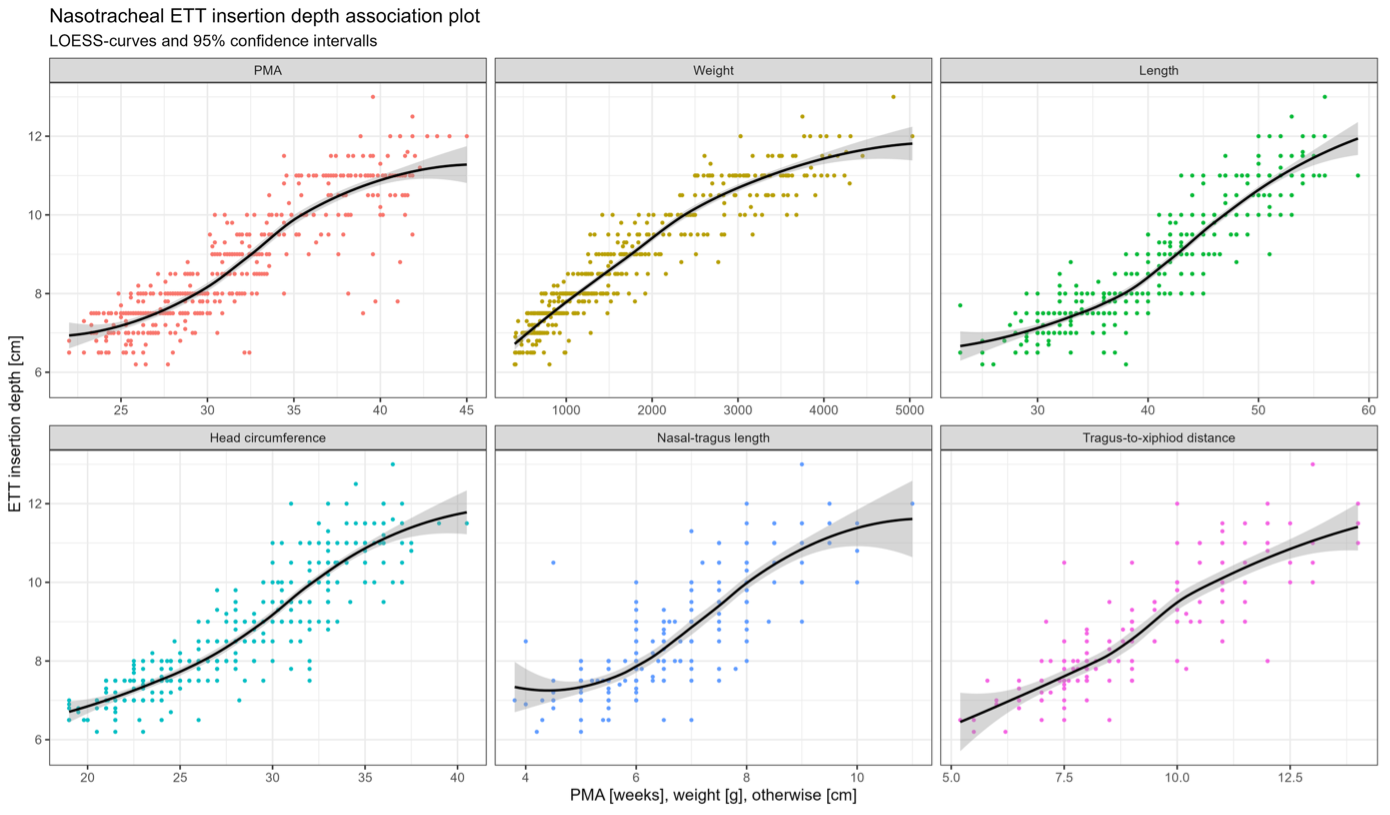

Supplement: Supplementary Figure S1 — Scatter plots showing the relationship between final nasotracheal ETT insertion depth and biometric parameters: postmenstrual age (PMA), weight, length, head circumference, nasal-tragus length, and tragus–to–xiphoid distance. LOESS smoothing curves illustrate the overall trends [file Image1.png]
