# Supplementary material for: Optimal nasotracheal tube insertion depth in neonates
Source: Front Pediatr. 2026 Feb 24;14:1770644. doi: 10.3389/fped.2026.1770644 (PMC12971967; doi:10.3389/fped.2026.1770644)
Supplement: Supplementary file 3 [file Table1.docx]

**Supplement Table 1** Performance comparison of linear regression models for predicting nasotracheal tube insertion depth.

| Name | Model | R2 | R2_adjusted | RMSE | Sigma | AIC_wt | AICc_wt | BIC_wt | Performance  Score |
| --- | --- | --- | --- | --- | --- | --- | --- | --- | --- |
| model_weight_normal | lm | 0.8894852 | 0.8892429 | 0.4938949 | 0.4949768 | 1.754574e-37 | 1.807363e-37 | 5.761800e-38 | 5.714286e-01 |
| model_weight | lm | 0.8835764 | 0.8833393 | 0.5169594 | 0.5180112 | 4.231023e-58 | 4.366574e-58 | 1.244113e-58 | 5.382006e-01 |
| model_TX | lm | 0.7251116 | 0.7238390 | 0.7348064 | 0.7382004 | 1.000000e+00 | 1.000000e+00 | 1.000000e+00 | 4.978219e-01 |
| model_length_normal | lm | 0.8610269 | 0.8606741 | 0.5553102 | 0.5567178 | 1.992352e-38 | 2.043733e-38 | 8.137818e-39 | 4.636892e-01 |
| model_HC_normal | lm | 0.8566659 | 0.8563002 | 0.5657629 | 0.5672043 | 6.760672e-41 | 6.933937e-41 | 2.782469e-41 | 4.460869e-01 |
| model_PMA_AGA | lm | 0.8514799 | 0.8511549 | 0.5723228 | 0.5735738 | 3.591643e-67 | 3.699919e-67 | 1.175598e-67 | 4.315398e-01 |
| model_length | lm | 0.8481563 | 0.8478262 | 0.5906219 | 0.5919044 | 1.314707e-74 | 1.354575e-74 | 4.261384e-75 | 4.071734e-01 |
| model_HC | lm | 0.8307792 | 0.8304097 | 0.6237399 | 0.6251003 | 9.877882e-85 | 1.017626e-84 | 3.222640e-85 | 3.461164e-01 |
| model_PMA | lm | 0.8019889 | 0.8015864 | 0.6751208 | 0.6764916 | 1.070950e-115 | 1.105316e-115 | 3.139523e-116 | 2.487421e-01 |
| model_ST | lm | 0.6980980 | 0.6970120 | 0.7626360 | 0.7653743 | 3.716682e-35 | 3.763795e-35 | 2.553307e-35 | 1.433398e-35 |

Model_weight_normal = model based on weight excluding weight measurements outside -2 – +2 SDS; model_weight = model based on all measured weights; model_TX = model based on tragus to xiphoid distance; model_length_normal = model based on length excluding length measurements outside -2 – +2 SDS; model_HC_normal = model based on head circumference excluding measurement outside -2 – +2 SDS; model_PMA_AGA = model based on post menstrual age including only appropriate for gestational age infants; model_length = model based on length; model_HC = model based on head circumference; model_PMA_AGA = model based on post menstrual age; model_ST = model based on sternum to tragus distance.
